# Misleading Epidemiological and Statistical Evidence in the Presence of Simpson's Paradox: An Illustrative Study Using Simulated Scenarios of Observational Study Designs

**DOI:** 10.25122/jml-2019-0120

**Published:** 2020

**Authors:** Chanapong Rojanaworarit

**Affiliations:** Department of Health Professions, School of Health Professions and Human Services, Hofstra University, Hempstead, New York, United States of America

**Keywords:** Simpson's Paradox, confounding variable, epidemiology, regression analysis, bias

## Abstract

This study empirically illustrates the mechanism by which epidemiological effect measures and statistical evidence can be misleading in the presence of Simpson's paradox and identify possible alternative methods of analysis to manage the paradox.

Three scenarios of observational study designs, including cross-sectional, cohort, and case-control approaches, are simulated. In each scenario, data are generated, and various methods of epidemiological and statistical analyses are undertaken to obtain empirical results that illustrate Simpson's paradox and mislead conclusions. Rational methods of analysis are also performed to illustrate how to avoid pitfalls and obtain valid results.

In the presence of Simpson's paradox, results from analyses in overall data contradict the findings from all subgroups of the same data. This paradox occurs when distributions of confounding characteristics are unequal in the groups being compared. Data analysis methods which do not take confounding factor into account, including epidemiological 2×2 table analysis, independent samples t-test, Wilcoxon rank-sum test, chi-square test, and univariable regression analysis, cannot manage the problem of Simpson's paradox and mislead research conclusions. Mantel-Haenszel procedure and multivariable regression methods are examples of rational analysis methods leading to valid results.

Therefore, Simpson's paradox arises as a consequence of extreme unequal distributions of a specific inherent characteristic in groups being compared. Analytical methods which take control of confounding effect must be applied to manage the paradox and obtain valid research evidence regarding the causal association.

## Introduction

**Figure 1:**
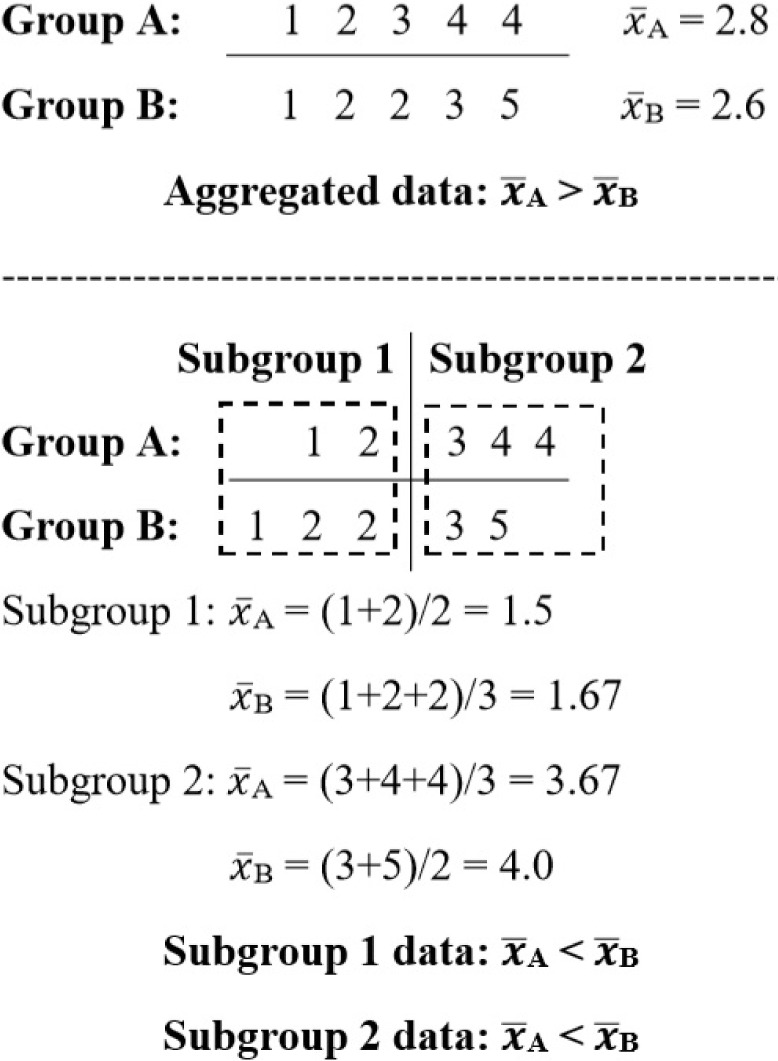
Numerical example of Simpson’s paradox.

A goal of epidemiology is to provide valid evidence regarding the determinant of health-related states in populations [1]. To achieve this goal, threats to validity of study such as confounding must be controlled through study design and appropriate analysis [2-5]. Confounding is a systematic difference of an inherent characteristic between groups being compared, which distorts a true association between exposure and outcome [3-4]. Simpson's paradox, an extreme form of confounding, is a phenomenon in which a paradox arises when crude analysis results obtained from aggregated data are opposite to the results in every mutually exclusive subgroups of the same data [6-7]. A simple numerical illustration of Simpson's paradox is provided in Figure 1. Given an example of two groups being compared, there are aggregated data of five numbers in each group. The average value () of these numbers in group A is higher than that of group B. Nonetheless, when the same data are stratified into two subgroups (1 and 2), the average values of numbers from group B become greater than those from group A in both subgroups. Thus, the analysis of aggregated data leads to a conclusion opposite to that suggested by mutually exclusive subgroups of the same data (Figure 1). This illustration exemplifies the need for careful interpretation of group differences determined in aggregated data since the underlying truth is paradoxical to the observed global difference.

In addition to Simpson's paradox, pitfalls in determining association also include using statistical methods that do not take confounders into the analysis and drawing inference about association based on group differences and statistical significance [8-10]. The Chi-square test, for example, is a method used to analyze the association between two categorical variables, and the result interpretation is based on the Chi-square statistic and p-value. [11] Nonetheless, since the test uses only information of two variables without considering other characteristics, confounding cannot be managed by this approach.

To empirically illustrate a mechanism by which Simpson's paradox arises, how it produces misleading epidemiological effect measures and statistical evidence, and the use of analysis methods which seem appropriate but incompetent to manage the paradox, this study provides three scenarios of Simpson's paradox in observational study designs. In each scenario, data are simulated and analyzed using different analytical approaches corresponding to each study design to show possible pitfalls in determining valid results and alternative analytical approaches to manage the problem.

## Material and Methods

Background, characteristics of data, and analytical methods are explained for each simulated scenario as followed.

### Scenario 1: An analytical cross-sectional study with a continuous outcome.

A research aim is to determine the difference in income between male and female dentists working in a particular state of the United States. A fictitious survey data of 240 dentists, 120 males, and 120 females, are generated. The monthly income is in United States dollars (USD). To illustrate Simpson's paradox, a variable of the ‘work sector’, either public or private, that each dentist works in is generated. The income data are generated non-normal in the aggregated and stratified datasets to simulate a situation when assumptions of normality of independent samples t-test are not satisfied, and the two-sample Wilcoxon rank-sum (Mann-Whitney) test is optional. Univariable and multivariable linear regression methods are also performed [12]. Results of regression methods are compared to those obtained from the t-test and Wilcoxon rank-sum test.

### Scenario 2: A cohort study with a dichotomous outcome

Let us suppose that a prospective cohort study is conducted to evaluate the preventive effect of influenza vaccination against influenza-related acute respiratory infection (ARI) among patients with chronic obstructive pulmonary disease (COPD). To simplify this illustration, ARI is measured as a dichotomous outcome, either occurring or not, within an assumed equal follow-up period for all patients. Patients receiving the vaccine are compared to those without vaccination. Since routine vaccine administration – exposure assignment – is based on the physician's judgment, patients with severe COPD are more likely to be indicated for vaccination. To exemplify this situation of confounding by indication [13-14] leading to Simpson's paradox, a variable of patient's COPD severity is generated and categorized into two categories of mild to moderate (low severity) versus severe to very severe (high severity), based on measurement of forced expiratory volume in one second (FEV1).

The cumulative incidence of ARI is calculated in the vaccine and non-vaccine groups. Effect measures, including risk difference (RD), risk ratio (RR), and vaccine effectiveness [(1-RR)×100%], are estimated [15]. Stratum-specific effect measures are calculated within each stratum of COPD severity and compared with corresponding effect estimates in the whole group. Mantel-Haenszel adjustment is performed to pool data across strata of COPD severity levels to obtain Mantel-Haenszel adjusted RR. The magnitude of confounding is calculated by [(RRcrude – RRadjusted) / RRadjusted] × 100%. RRadjusted is used as the denominator in the formula due to the epidemiological reason that the adjusted effect is unconfounded and considered as the starting measure in the calculation. Regression methods are additionally employed. A generalized linear model for the binomial family with an identity link is applied to estimate RD [16]. Poisson regression with robust standard errors is applied to estimate RR [17-18]. Differences in crude and adjusted effect measures from all of these analyses are discussed.

### Scenario 3: A case-control study with a dichotomous outcome

Coffee consumption has been hypothesized to be associated with lung cancer [19]. Let us suppose that a hospital-based case-control study is undertaken to evaluate the hypothesis. Study participants include 250 lung cancer cases and 250 controls. The controls are patients whose admission diagnoses are not likely related to the exposure of interest [20]. To simplify the illustration, coffee consumption is generated as a dichotomous variable as <1 cup a day (reference group) or ≥1 cup a day (index group). To exemplify Simpson's paradox, the smoking status –smoker versus non-smoker– is generated as a confounder.

The odds ratio (OR) is calculated to measure the association [21-22]. Stratum-specific OR is calculated for each stratum of the smoking status. The mantel-Haenszel procedure is performed to obtain Mantel-Haenszel adjusted OR [23]. The magnitude of confounding is calculated by [(ORcrude – ORadjusted) / ORadjusted] × 100%. Univariable and multivariable analyses using binary logistic regression are additionally performed to estimate crude and adjusted ORs. Different OR estimates from these different approaches are discussed.

The α value of 0.05 is specified for all statistical hypothesis tests in all scenarios.

## Results

### Scenario 1

In the aggregated data, the assumption of normality for the t-test to compare incomes is evaluated using the Shapiro-Wilk test, which indicates non-normal distributions of incomes in both sexes (p<0.001). The two-group variance-comparison test shows a non-significant difference between variances of incomes in both sexes (p=0.693), allowing equal variance assumption for the t-test. The t-test with equal variances shows that, regardless of normality assumption violation, female dentists have a significantly higher average income.

In contrast, the Wilcoxon rank-sum test shows no statistical evidence of the difference in monthly incomes. In both subgroups of dentists by the working sector, the normality test indicates non-normal distributions of incomes in both sexes. Variances of incomes in both sexes cannot be assumed equal, as shown by the two-group variance-comparison test (p<0.05). When a t-test with unequal variances is applied, irrespective of normality assumption violation, it indicates significantly higher average incomes among males in both working sectors, contradicting the finding in the aggregated data. The Wilcoxon rank-sum test indicates that male and female dentists have different distributions of incomes in the population of dentists working in each sector. Univariable linear regression reveals a significantly lower average income of male dentists, similar to the result from the t-test applied to aggregated data.

Nonetheless, after adjusting for the difference by working sectors, the multivariable model contrastively identifies a significantly higher average income among male dentists. It is noted that, from post-estimation model diagnostics, the distribution of standardized regression residuals is not normal, as determined by the Shapiro-Wilk test (p=0.022), indicating a violation of normality assumption of linear regression analysis (Table 1).

**Table 1:** Average monthly incomes of dentists by gender and linear regression analyses of difference in average monthly incomes by gender (N=240).

	**Male**	**Female**	**p-value**
**Overall**	n = 120	n = 120	
Mean ± SD	19,111.3 ± 8,780.2	21,722.3 ± 8,466.9	0.020^†^
Median (IQR)	13,516.7 (18,208.3)	26,166.7 (16,510.0)	0.451*
Min. – Max.	9,033.3 – 33,266.7	9,250 – 32,666.7	
**Subgroups**			
**Public**	n = 75 (62.5%)	n = 45 (37.5%)	
Mean ± SD	12,648.7 ± 2,368.2	11,143.8 ± 1,555.7	<0.001^‡^
Median (IQR)	12,700.0 (1,966.7)	11,333.3 (3,006.7)	<0.001*
Min. – Max.	9,033.3 – 20,000	9,250 – 13,333.3	
**Private**	n = 45 (37.5%)	n = 75 (62.5%)	
Mean ± SD	29,882.2 ± 3,037.3	28,069.3 ± 2,228.1	<0.001^‡^
Median (IQR)	31,000.0 (5,400.0)	28,186.7 (3,150.0)	<0.001*
Min. – Max.	23,366.7 – 33,266.7	24,666.7 – 32,666.7	

	**Univariable linear regression**			**Multivariable linear regression**		
	Coefficient	95% CI	p-value	Coefficient	95% CI	p-value
**Gender**						
Female		Reference			Reference	
Male	-2,611	-4,804.5 -417.5	0.020	1,658.9	1,044.6 2,273.2	<0.001
**Sector**						
Public		-			Reference	
Private		-		17,079.56	16,465.3 17,693.8	<0.001
**Constant**	21,722.3	20,171.2 23,273.3	<0.001	11,047.53	10,478.1 11,617.0	<0.001

SD, standard deviation; IQR, interquartile range; Min., minimum; Max., maximum;

CI, confidence interval; %, percentage by column

^†^Independent samples t-test with equal variances

^‡^Independent samples t-test with unequal variances

*Two-sample Wilcoxon rank-sum test.

### Scenario 2

In the aggregated data, ARI incidence is higher among COPD patients receiving the influenza vaccine. This indicates the ineffectiveness of the vaccine against ARI. In contrast, ARI incidences observed in both subgroups of patients by COPD severity levels are lower among vaccinated patients. The lower ARI incidence among vaccinated patients in each subgroup enables the calculation of crude vaccine effectiveness [15]. The crude vaccine effectiveness in reducing ARI incidence is 50% and 17% in patients with low and high COPD severity, respectively. RR exceeding one, indicating a higher risk of ARI in the vaccine group, is observed in the aggregated data. However, RRs observed in both subgroups are contrastively less than one, indicating the lower risk of ARI in vaccinated patients. These stratum-specific RRs of 0.5 and 0.83 are not significantly different, as indicated by the Mantel-Haenszel test of homogeneity (p=0.169) and can be pooled to obtain Mantel-Haenszel adjusted RR of 0.73, which determines the lower risk of ARI in vaccinated patients after adjusting for the difference in COPD severity between groups. The magnitude of confounding by COPD severity on the association between influenza vaccine and ARI is 63% (Table 2).

**Table 2:** Influenza-related acute respiratory infection in the overall groups and COPD severity subgroups in patients with and without influenza vaccination (N=320).

	Acute respiratory infection	p-value^‡^	Incidence	RD	RR	Effectiveness [1-RR]×100 (%)
	Yes n (%)^†^	No n (%)^†^					
Overall							
Vaccine	76 (47.5)	84 (52.5)	0.176	0.48	0.08	1.19	N/A
No vaccine	64 (40.0)	96 (60.0)		0.40			
Subgroups:							
Low severity*							
Vaccine	6 (15.0)	34 (85.0)	0.062	0.15	- 0.15	0.50	50
No vaccine	36 (30.0)	84 (70.0)		0.30			
High severity*							
Vaccine	70 (58.3)	50 (41.7)	0.190	0.58	- 0.12	0.83	17
No vaccine	28 (70.0)	12 (30.0)		0.70			
**M-H adjusted RR**						0.73	27
**M-H test of homogeneity (p-value)**						0.169	
Magnitude of confounding (%)**						63.0	

RD, risk difference; RR, risk ratio; N/A, not applicable

M-H adjusted RR, Mantel-Haenszel adjusted RR

M-H test of homogeneity, Mantel-Haenszel test of homogeneity of stratum-specific RRs

^†^Percentage by row

^‡^Chi-square test

*Severity of COPD

**Calculated by [(RRcrude – RRadjusted) / RRadjusted]×100%.

The univariable regression method estimates crude RD at 0.08, which is the same value obtained from the 2×2 table analysis in Table 2. The positive value of RD indicates higher ARI risk or ineffectiveness in the vaccine group, though not statistically significant. RD adjusted for COPD severity at - 0.14 in the multivariable model contrastively determines the preventive effect of the vaccine. A crude RR of 1.19 from the univariable model indicates no effect of the vaccine on ARI risk. The crude RR is the same value as that calculated from the 2×2 table in Table 2. In contrast, after controlling the effect of COPD severity, the adjusted RR of 0.74 indicates the preventive benefit of the vaccine. The adjusted RR obtained from the multivariable model is the same as that previously derived from the Mantel-Haenszel procedure in Table 2 (Table 3).

**Table 3:** Univariable and multivariable analyses of influenza-related acute respiratory infection; risk difference and risk ratio in COPD patients with and without influenza vaccination (N=320).

	**Univariable analysis**			**Multivariable analysis**		
	**crude**	**95% CI**	**p-value**	**adjusted**	**95% CI**	**p-value**
	**Risk difference**			**Risk difference**		
**Vaccine**						
No		Reference			Reference	
Yes	0.08	- 0.03, 0.18	0.175	- 0.14	- 0.24, - 0.03	0.012
**Severity^†^**						
Low		-			Reference	
High		-		0.42	0.31, 0.53	<0.001
		**Risk ratio**			**Risk ratio**	
**Vaccine**						
No		Reference			Reference	
Yes	1.19	0.92, 1.53	0.179	0.74^‡^	0.59, 0.94	0.015
**Severity**						
Low		-			Reference	
High		-		2.70	2.01, 3.64	<0.001

CI, confidence interval

^†^Severity of COPD

^‡^Vaccine effectiveness adjusted for severity of COPD is 26% [from (1- RRadjusted)×100%].

### Scenario 3

In the aggregated data, the odds of drinking ≥ 1 cup of coffee per day is approximately two times among lung cancer cases compared to controls. However, ORs and p-values determined in the subgroups by smoking statuses contradict the previous finding. The stratum-specific values of OR in both subgroups are close to one, indicating no association. These stratum-specific ORs are not significantly different as determined by the test of homogeneity (p=0.949) and can be pooled by the Mantel-Haenszel method to obtain an adjusted OR of 1.12. The magnitude of confounding by smoking is 87.5% (Table 4).

**Table 4:** Coffee consumption and lung cancer in the overall groups and smoking status subgroups, as well as logistic regression analyses of the association (N=500).

	Lung cancer		Odds ratio (OR)	p-value^‡^
	Yes [n=250] n (%)^†^	No [n=250] n (%)^†^		
**Overall**				
Coffee (+)	200 (80.0)	164 (65.6)	2.10	<0.001
Coffee (–)	50 (20.0)	86 (34.4)		
**Subgroups:**				
**Non-smokers**				
Coffee (+)	24 (64.9)	125 (61.9)	1.14	0.731
Coffee (–)	13 (35.1)	77 (38.1)		
**Smokers **				
Coffee (+)	176 (82.6)	39 (81.2)	1.10	0.821
Coffee (–)	37 (17.4)	9 (18.8)		
**M-H adjusted OR**			1.12	
**M-H test of homogeneity (p-value)**			0.949	
Magnitude of confounding (%)*			87.5	

	**Univariable analysis**			**Multivariable analysis**		
	**cOR**	**95% CI**	**p-value**	**aOR**	**95% CI**	**p-value**
**Coffee**						
<1 cup/day		Reference			Reference	
≥1 cup/day	2.10	1.40, 3.15	<0.001	1.12	0.65, 1.92	0.683
**Smoking**						
No		-			Reference	
Yes		-		23.72	14.69, 38.31	<0.001

Coffee (+), ≥ 1 cup a day; Coffee (–), <1 cup a day

CI, confidence interval; M-H adjusted OR, Mantel-Haenszel adjusted OR;

M-H test of homogeneity, Mantel-Haenszel test of homogeneity of stratum-specific ORs;

cOR, crude odds ratio; aOR, adjusted odds ratio

^†^ Percentage by column

^‡^ Chi-square test

* Calculated by [(OR_crude_ – OR_adjusted_) / OR_adjusted_]×100%

The univariable logistic regression method provides a crude OR of 2.1 and statistical significance, which is similar to the findings from the 2×2 table analysis and Chi-square test. Contrastively, multivariable logistic regression revealed an OR adjusted for the effect of smoking to be close to one, showing no association between the designated level of coffee drinking and lung cancer. A non-significant p-value for this association is also obtained. The adjusted OR from the multivariable model is the same as the ones from the Mantel-Haenszel procedure (Table 4).

## Discussion

In all scenarios, the effect measures obtained from the analysis of aggregated data lead to conclusions that contradict the ones suggested by the results in subgroups of the same data. These different pieces of evidence indicate that Simpson's paradox can occur in various types of data (continuous and categorical data) and effect measures (mean difference, RD, RR, and OR).

In general, a variable or characteristic that inherently exists in data can be a confounding factor for a studied association when such variable is (1) associated with the outcome, (2) unequally distributed across exposure groups being compared, and (3) not an effect of the exposure or part of the causal pathway between exposure and outcome [3]. Simpson's paradox, a form of severe confounding problem, usually arises from uneven distribution of the confounding factor among groups being compared [6, 24]. In scenario 1, the analysis of mean in aggregated data reveals a significantly higher average income among female dentists. Without information regarding the working sector, a confounder, observed mean difference, and statistical significance would lead to such a conclusion. Nonetheless, taking a confounder into account, the difference in average incomes between sexes can be alternatively explained by unequal proportions of dentists working in public and private sectors in the female and male groups. All dentists in the private sector earn even more than the highest income in the public ones. Since female dentists mostly work in the private sector (62.5%) while only 37.5% of the males do, the imbalance results in higher average income among females. Contrastively, when aggregated data are stratified into two subgroups, higher mean incomes among males are determined in both subgroups (Table 1). Adding an extra dimension of the working sector to the data enables the detection of a subtler difference in average income between sexes. However, a confusing paradox occurs, and the answer to whether there is gender discrimination in incomes becomes inconclusive.

One may argue that independent samples t-test should be avoided due to normality assumption violation, and the two-sample Wilcoxon rank-sum test should instead be employed. [25] Nonetheless, the Wilcoxon rank-sum test also leads to paradoxical findings in the aggregated and subgroup data. Although the Wilcoxon rank-sum test is commonly used as an alternative to the t-test when data are non-normal and small (n < 30), the test compares neither means nor medians [26]. It actually tests ‘mean ranks’, which is not the same thing as medians, and it is possible to have two datasets with identical medians but statistically significant Wilcoxon rank-sum test results. Therefore, using the Wilcoxon rank-sum test to answer the question about the comparison of means can mislead the conclusion [26-29].

Regarding normality assumption violation, the robustness of the t-test has been demonstrated that it is still valid in the analysis of non-normal data [25, 27, 30] and extremely small sample sizes as long as the effect size is expected to be large [29, 31]. Above all, the t-test and rank-sum test do not take a confounder into account and are incapable of managing the paradox.

Univariable linear regression provides results similar to that from the t-test applied to aggregated data. This similarity occurs as the univariable model does not know the information about the working sector. Multivariable linear regression analyzing both sexes and the working sector simultaneously, taking a confounder into account, provides a valid answer: independent of working sectors, male dentists earn significantly higher incomes than females. The multivariable analysis determines the effect of each variable on the outcome, independent of the other variables [8]. As previously noted, applying multivariable linear regression in this scenario violates regression assumption as standardized regression residuals are not normally distributed. Therefore, evidence from analyses in both subgroups and multivariable models should be holistically considered as confounder is considered in these analyses. It is noted that, although the analytical cross-sectional study is not a rigorous design to determine the temporal relationship between exposure and outcome, confounding factors should still be controlled to allow a fair comparison of outcome between exposure groups as exemplified in this scenario.

Scenario 2 illustrates how Simpson's paradox misleads RD and RR. In the aggregated data, the estimated RD>0 and RR>1 indicate the vaccine's ineffectiveness. Higher ARI incidence in the vaccine group is caused by an unfair comparison, as most vaccinated patients have high COPD severity (n=120, 75%), while this is the case for only 25% of those without the vaccine. ARI is more likely to develop among patients with high COPD severity, as ARI occurs in 98 of 160 (61.3%) patients with high severity but only occurs in 42 of 160 (26.3%) patients with low severity (Table 2). The cause of unequal proportions of patients with different COPD severity in the vaccine and non-vaccine groups is the indication for vaccination. The vaccine is more likely to be prescribed for patients with high COPD severity who are at greater risk of influenza. This ‘confounding by indication’ can occur when observational studies are applied to evaluate the efficacy of interventions [13-14]. In contrast, lower ARI incidence among vaccinated patients is determined in each subgroup. Vaccine effectiveness can also be indicated by RD<0, RR<1, and preventive effectiveness (%). This contradictory evidence from the aggregated and subgroup data indicated Simpson's paradox, which leads to indecisiveness about vaccine effectiveness. This scenario also serves as an example when confounding by indication can lead to Simpson's paradox. Besides, the non-significant p-values consistently obtained from the chi-square test in the aggregated and subgroup data can mislead to the conclusion that there is no association between vaccine and ARI as the observed epidemiological measures are likely to occur by chance alone (Table 2).

To manage the paradox, 'stratified analysis’ is applied by stratifying aggregated data into subgroups by COPD severity, thus enabling a fair comparison of ARI incidences between the vaccine and non-vaccine groups for each level of COPD severity [23]. The Mantel-Haenszel test of homogeneity is applied to determine that stratum-specific RRs are not significantly different and can be pooled to obtain the single summary of adjusted RR of 0.73, which indicates the vaccine's preventive effect (Table 2). This evidence leads to a valid conclusion that the vaccine has a preventive effect against ARI. Although Mantel-Haenszel procedure can be applied to obtain an estimate of association adjusted for the effect of one or several confounders, controlling multiple confounders requiring stratification of data into strata with smaller data can be problematic [23]. Thus, the more practical approach of regression analysis is applied to adjust the effect from multiple confounders [32-33]. RD and RR obtained from univariable regression are similar to those obtained from the 2×2 table in Table 2. These estimates are thus regarded as crude estimates of the association.

In contrast, multivariable regression analyses reveal statistically significant RD<0 and RR<1 adjusted for confounding, indicating the preventive effect of the vaccine (Table 3). Evidence obtained from multivariable regression analysis, including the magnitude and direction of the association, interval estimate of effect (95% confidence interval), and p-value, should be comprehensively considered to reach a valid conclusion.

Scenario 3 further exemplifies Simpson's paradox in dichotomous outcomes in the context of a case-control study in which OR is the effect measure. Without information on the smoking status, a confounder, OR of 2.1, and statistical significance from the chi-square test in aggregated data would mislead to the conclusion that coffee drinking increases the odds of developing lung cancer. This false conclusion is possible due to the uneven distribution of smokers in the groups being compared, a larger proportion of smoking inherently exists among coffee drinkers. The clinically-meaningful OR and statistical significance obtained from the analysis of aggregated data do not ensure the absence of Simpson's paradox. A confusing paradox still occurs as the OR and p-value obtained from analysis in each subgroup by smoking status indicate no association between coffee drinking and lung cancer. Mantel-Haenszel procedure and multivariable logistic regression consistently indicate the same evidence of adjusted OR of 1.12 and non-significant p-value, which lead to the valid conclusion of no association between coffee drinking and lung cancer. It is also important to note that a variable qualifying as a potential confounder must be considered according to the context of the study. For example, if this study in scenario three is conducted in a particular religious community where smoking is prohibited, smoking does not qualify to be a confounder in such a case.

In each scenario, only one confounder is considered. Nonetheless, in reality, the exposure-outcome association can still be the other way round when more potential confounders are included in analysis. Therefore, potential confounders should always be carefully identified and controlled to avoid confounding bias.

## Conclusion

Simpson's paradox arises as a consequence of extreme unequal distributions of confounders in groups being compared. To avoid the statistical illusion and misleading effect measure, analytical approaches that are capable of controlling the confounding effect must always be employed to obtain a valid measure of a causal association.

## Conflict of Interest

The authors declare that there is no conflict of interest.
